# Some Confirmed Symptoms

**Published:** 1891-05

**Authors:** J. R. Haynes

**Affiliations:** Indianapolis, Ind.


### SOME CONFIRMED SYMPTOMS.

J. R. Haynes, M. D., Indianapolis, Ind.

(Read at the Twenty-fourth Annual Session of the Indiana Institute of Homœopathy, Indianapolis, May 15th, 1890.)

### Belladonna.

Mrs. ——, about forty-eight years old; light complexion; fat, would weigh about one hundred and ninety pounds; dark brown hair; blue eyes; at the same time very nervous and fidgety; said she could never get well—could not live twenty-four hours; very excitable, must shed tears; aggravated by motion. Headache as if some hard substance was pressing against the forehead; external head extremely sensitive to touch; eyes sensitive to light, painful, with a deep-seated, dull pain in the back of the eyeballs; eyes very sensitive to touch. Fluent coryza from the nose; frequent sneezing; face pale, slightly mottled; tongue coated white and sticky; anterior papilæ red and prominent; gummed-up taste in the mouth; mouth dry with but little thirst. Throat feels raw, dry, and sore; continuous hawking to clear out the sticky mucus; swallowing slightly painful; fauces bright red and sore to touch; craves lemonade, or "something to cut the phlegm." Suppressed or incomplete eructations, with spasmodic cramp in the stomach. Region of the liver painful and sore to touch; great tenderness to slight pressure over the whole abdomen; abdomen distended with gas. Frequent desire to pass small quantities of rather pale, watery urine; the bladder must be relieved at once, or the urine would dribble away from relaxed sphincter. Breathing short, hurried, and anxious; pulse quick (110), full, but rather soft. Every muscle in the body extremely sore, the slightest movement or touch very painful; must stay on her back, as all other positions were excruciatingly painful; any attempt to sleep would cause a spasmodic start or jerking of the whole body like an electrical shock, which was extremely painful, and caused her to scream out with pain. The whole body covered with a hot, drenching perspiration, which caused great restlessness; seemed to aggravate all of the other symptoms, yet wanted to be heavily covered. Perspiration like hot water; skin of normal color, or as natural when in perspiration, which was continuous.

℞ Belladonna$^{10m}$ in water, one teaspoonful every two hours, to be kept up for twenty-four hours. The next visit found some improvement; not so much sweating; muscles not so sore; could move without so much pain; beginning to want food; had slept some, felt refreshed on waking. ℞ Sac-lac. in water every two hours. Third visit, still improving; Sac-lac. as above. Fourth visit, better in every way; Sac-lac. The fifth visit found her dressed and sitting up; continued Sac-lac. for several days. There has been no return since.

### Rhus-tox.

Mrs. ——, aged twenty-two years; light complexion, medium height; dark auburn hair; would weigh about one hundred and twenty pounds. Rather lazy and desponding disposition, always looking on the gloomy side and ready to meet trouble more than half-way; the mother of one child about six months old. Had been on the street in a cold, drizzling rain, got her feet damp; thought little of it, and neglected to change her clothing. I found her in bed, very low-spirited and ready to weep; said she would never get well again. Very restless; symptoms all aggravated by rest; vertigo in the forehead and nausea upon rising. Pulse, 110; temperature, 102°. Wrists, elbows, shoulder, ankle, and knee joints swollen, red, and very sore to touch. Putrid, sticky taste in the mouth, with little thirst; no appetite; tough mucus in the throat, which caused nausea; urine scanty, dark brown, with smarting when voiding it, which immediately ceased with the evacuation. Stitches in the intercostal muscles, worse by rest or when commencing to move, but after movement felt better for a short time; stiffness in the neck and shoulders; spasmodic yawning, but could not sleep. Some itching of the skin, relieved by rubbing, no eruption discernible.

℞ Rhus-tox.$^{10m}$ in water, one teaspoonful every two hours for the first twenty-four hours.

The next visit found a decided improvement; had slept a portion of the night; could remain quiet; not so gloomy; believed she would get well. Sac.-lac. in water every two hours.

The third visit found her up and dressed, looking after her household.

Sac-lac. for four or five days; discharged. No complaint afterward.

### Mercurius-iod. et Kali-iod.

Some years since I saw this compound remedy recommended in some of the journals as an excellent remedy in cases of acute catarrhal fevers. I procured some of it in the sixth trituration, but had to send to several pharmacies before getting it. A short time afterward Mrs. —— sent for me. She had a violent attack of catarrhal fever; dull, heavy frontal headache, not aggravated or ameliorated by motion; felt stupid; irritating water running from the eyes; free, watery discharge from the nose; tongue coated white; the whole pharynx of a purplish red; painful deglutition. Throat, fauces, and mouth filled with catarrhal mucus; gummed-up, sticky taste in the mouth; soreness of the muscular fibres of the whole body. Pulse, 90; skin hot and dry; paroxysms of coughing, must sit up to cough, which sounded hoarse; some rattling on inhaling, with a discharge of considerable yellowish, frothy sputum which gave but little relief.

Merc-iod. et Kali-iod.$^{6}$, about one grain in a half glass of water, one teaspoonful every two hours for the first twenty-four hours. At the next visit found my patient up and dressed, feeling much better. Left Sac-lac. for several days, with request to be informed immediately should any relapse occur. The Sac-lac. completed a perfect cure.

Did not see another case for over a year, when I had a number with almost the above identical symptoms, and all were cured in the same manner.

Some patients came to the office with these catarrhal symptoms and were given powders for three or four days, and, so far as I know, every one who took the remedy for more than twenty-four hours was made a great deal worse. All of the above symptoms were severely aggravated, and would take a number of days for their relief as well as the selection of other remedies. Perhaps the aggravations would have passed off with a cure if we could have waited long enough, but their sufferings were so severe we considered it too dangerous to wait for the secondary action.

During the past winter in the epidemic of influenza which has prevailed, almost every case which I saw (if they came before dosing themselves) had more or less the above identical symptoms, which the above remedy would quickly relieve and completely cure, unless they would expose themselves and take a fresh cold, when a repetition of Merc-iod. et Kali-iod. would be worse than useless.

For these relapses I found Rhus-tox. or Dulcamara the remedy, according to the symptoms which were produced at the time.

The above symptoms have been verified hundreds of times in my cases, both of Belladonna, Rhus, and Merc-iod. and Kali-iod. In numerous instances during the past winter a single dose dry on the tongue has made a complete and quick cure. I think one dose acts more quickly and more deeply, as well as making a more perfect cure than by repeating, and is not so liable to cause a relapse or change of symptoms. I hope such of you as have not tried this last remedy will give it a fair test according to the above symptoms, and then publish all of your failures in all of the journals. I would recommend a higher potency, and shall procure it as soon as convenient.

